# A Canadian Advanced Physiotherapist Practitioner Shared-Care Model in Pediatric Rheumatology Offers Safe and Quality Care in the Management of Juvenile Idiopathic Arthritis—Comparing Key Performance Indicators with the PR-COIN Registry

**DOI:** 10.3390/children12121675

**Published:** 2025-12-10

**Authors:** Julie Herrington, Patrick Clarkin, Jade Singleton, Karen Beattie, Sheetal S. Vora, Katelyn Banschbach, Catherine A. Bingham, Tania Cellucci, Danielle Fair, Mileka Gilbert, Beth Gottlieb, Julia G. Harris, Liane Heale, Tzielan Lee, Melissa L. Mannion, Edward J. Oberle, Nancy Pan, Jonathan Park, Mary Toth, Jennifer E. Weiss, Michelle Batthish

**Affiliations:** 1 McMaster Children’s Hospital, Hamilton Health Sciences, Hamilton, ON L8S 1C7, Canada; 2 School of Rehabilitation Science, McMaster University, Hamilton, ON L8S 1C7, Canada; 3 Integrated Biomedical Engineering & Health Sciences, McMaster University, Hamilton, ON L8S 4L8, Canada; 4 Research Institute, Seattle Children’s Hospital, Seattle, WA 98101, USA; 5 Division of Pediatric Rheumatology, Department of Pediatrics, McMaster University, Hamilton, ON L8S 4K1, Canada; 6 Atrium Health Levine Children’s, Wake Forest School of Medicine, Charlotte, NC 28203, USA; 7 School of Medicine, Wake Forest University, Winston-Salem, NC 27101, USA; 8 Department of Pediatrics, Cincinnati Children’s Hospital Medical Center, Cincinnati, OH 45229, USA; 9 Department of Pediatrics, Division of Pediatric Rheumatology, Penn State Children’s Hospital, Hershey, PA 17033, USA; 10 Department of Pediatrics, Division of Rheumatology, Medical College of Wisconsin, Children’s Wisconsin, Milwaukee, WI 53226, USA; 11 Department of Pediatrics, Division of Rheumatology, Medical University of South Carolina, Charleston, SC 29425, USA; 12 Pediatric Rheumatology, Northwell, Cohen Children’s Medical Center, New Hyde Park, NY 11040, USA; 13 Department of Pediatrics, Children’s Mercy Kansas City, Kansas City, MO 64108, USA; 14 School of Medicine, University of Missouri-Kansas City, Kansas City, MO 64108, USA; 15 School of Medicine, Stanford University, Stanford, CA 94304, USA; 16 Department of Pediatrics, University of Alabama at Birmingham, Birmingham, AL 35233, USA; 17 Department of Pediatrics, Division of Rheumatology, Nationwide Children’s Hospital, Columbus, OH 43205, USA; 18 Department of Pediatrics, Division of Rheumatology, The Ohio State University, Columbus, OH 43210, USA; 19 Department of Medicine, Division of Pediatric Rheumatology, Hospital for Special Surgery, New York, NY 10021, USA; 20 Department of Pediatrics, London Health Sciences Centre, London, ON N6A 5W9, Canada; 21 Pediatric Rheumatology, Nemours Children’s Hospital, Orlando, FL 32827, USA; 22 Department of Pediatrics, Hackensack University Medical Center and Hackensack Meridian Health, Hackensack, NJ 07601, USA

**Keywords:** advanced practice physiotherapy, juvenile idiopathic arthritis, pediatric rheumatology, models of care, safety, quality indicators, learning health network

## Abstract

**Highlights:**

**What are the main findings?**

- The pediatric rheumatology Advanced Physiotherapist Practitioner (APP) Shared-Care Model (SCM) met or exceeded Pediatric Rheumatology Care and Outcomes Improvement Network (PR-COIN) performance goals for key quality indicators in juvenile idiopathic arthritis (JIA) care.
- APP SCM key performance indicator documentation of disease activity and safety monitoring was comparable to standard care delivered by pediatric rheumatologists at the Same Center and across the PR-COIN network.

**What are the implications of the main findings?**

- The APP SCM provides high-quality and safe care for children with JIA, supporting the effectiveness of advanced practice physiotherapists in collaborative pediatric rheumatology models.
- Expanding and replicating APP SCMs across pediatric rheumatology centers may improve access and maintain high standards of care nationwide.

**Abstract:**

**Background/Objectives**: Canadian Advanced Physiotherapist Practitioner (APP) roles have existed for over 25 years in pediatric rheumatology. The APP can manage many common pediatric rheumatic conditions most often in Shared-Care Models (SCMs) with pediatric rheumatologists (PRs). The quality of care children receive in an APP SCM compared to traditional care is unknown. The Pediatric Rheumatology Care and Outcomes Improvement Network (PR-COIN) tracks quality measures as Key Performance Indicators (KPIs) in juvenile idiopathic arthritis (JIA) care. This study aimed to analyze the frequency of KPIs documented in a pediatric rheumatology APP SCM from a single center and compare to PR-COIN’s performance targets to assess the quality and safety of care. **Methods**: A retrospective chart review of JIA cases managed in a pediatric rheumatology APP SCM over a 2-year period was conducted. KPIs for disease activity, safety monitoring and access to care were evaluated. Frequency of KPI documentation by the APP were compared to target performance goals (≥40, ≥70 or ≥80% documentation rate depending on KPI) and with PR-COIN data from the Same Center (SC) (three rheumatologists) and PR-COIN (15 centers). **Results**: Documented KPIs were compared between the APP SCM, SC and PR-COIN registry (138; 140; 11,431 eligible visits, respectively) between June 2022–May 2024. Demographics were similar between groups. Increased percentages of patients with polyarticular rheumatoid factor positive and psoriatic subtypes were seen by APP compared to SC and PR-COIN. Documentation frequency of all disease activity and safety monitoring KPI performance goals were either higher in the APP SCM or comparable to SC and PR-COIN. **Conclusions**: The pediatric rheumatology APP SCM exceeded PR-COIN performance goals for KPI documentation, establishing a high level of quality and safety of care for children with JIA when managed in this model of care. Next steps include replicating this study in other pediatric rheumatology centers with an APP SCM.

## 1. Introduction

Advanced Practice Physiotherapy has evolved worldwide over the past three decades. Canada, along with 13 other countries, currently recognizes the Advanced Physiotherapist Practitioner (APP) role [1]. World Physiotherapy has described Advanced Practice Physiotherapy as “a higher level of practice that requires advanced analytical skills, knowledge, attitudes and clinical reasoning” [2]. APP models of care (MOC) have demonstrated increased access to care, decreased wait times, and increased patient satisfaction [3,4,5]. APPs perform extended-scope tasks including triage, assessments and management of complex musculoskeletal (MSK) cases, tasks traditionally completed by physicians [6,7]. These roles have been primarily implemented in MSK-focused areas of health care including rheumatology, orthopedics, emergency departments, and primary care [8].

In Canada, APP MOC were first described in 1995 as a novel way to increase access to care in pediatric rheumatology [9,10]. In 2005, the “Advanced Clinician Practitioner in Arthritis Care” (ACPAC) training program was established, modeled after this initial role in pediatric rheumatology [7,10]. ACPAC training involves a 10-month post-licensure, competency-based program that develops advanced rheumatology and orthopedic knowledge, skills, and attitudes for clinicians to assume extended-scope roles in the health care system [11,12]. The ACPAC program has trained nearly 150 experienced physiotherapists, occupational therapists, nurses, and chiropractors for extended-scope roles in rheumatology and MSK care [13]. Many ACPAC clinicians are employed in the Canadian health care system under the job title of an APP, typically working in extended-scope Shared-Care Models (SCMs) with physician partners to improve access to care [7,13,14].

Pediatric rheumatology is currently experiencing a physician workforce shortage in Canada [15]. Mounting pressures of increasingly complex caseloads combined with a diminishing workforce have the potential to negatively impact the quality of care for children with rheumatic diseases [16]. To address this concern, APP SCMs using ACPAC clinicians have been implemented in some pediatric rheumatology centers across Canada [7,17]. The APP assesses new patients whose referrals are MSK-focused, orders and interprets investigations, provides a diagnosis, and manages cases long-term. Up to 80% of new consults seen in an APP SCM in pediatric rheumatology have been shown to have a non-rheumatic disease [18]. For the remaining 20% diagnosed with a rheumatic disease, the APP becomes their ongoing primary practitioner in a SCM with a physician, allowing for increased physician caseload capacity [18]. However, there is a lack of evidence that examines the quality of care, safety and efficacy of disease outcomes when children with rheumatic diseases are followed long-term in an APP SCM.

**Figure 1:**
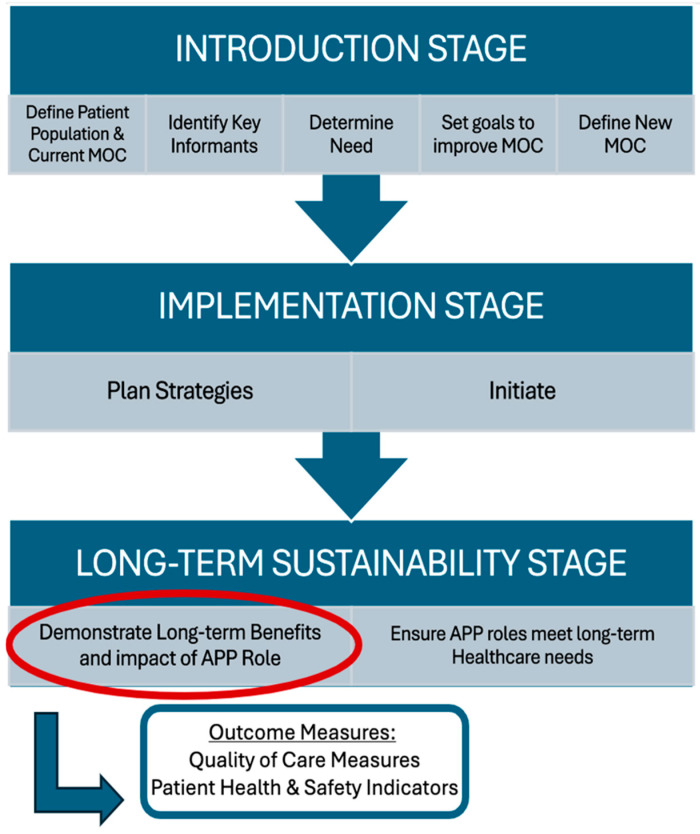
Adaptation of PEPPA Plus Framework for Advanced Physiotherapist Practitioner (APP) Role Development with focus on demonstrating long-term benefits by measuring quality and safety of care [20,21]. MOC—Model of Care; PEPPA—Participatory, Evidence-informed, Patient-centered Process for APN Role Development.

The nursing profession has emerged as a leader in the development of advanced practice roles, establishing effective pathways and steps for successful role implementation. The PEPPA (Participatory, Evidence-informed, Patient-centered Process for APN role development) framework is a systematic, health care planning framework designed to support the development, implementation, and evaluation of advanced practice nursing roles [19]. (Figure 1) The framework has been successfully used with the development of a Canadian APP role to improve access and quality of care for patients requiring hip and knee replacement surgery [20,21]. Much of the APP research to date has focused on the introductory and implementation stages of this framework, with less emphasis on long-term sustainability. With the exponential growth of APP roles in Canada, where long-term management of chronic MSK diseases is becoming more prevalent, the quality of care, safety and efficacy must be established [7].

Key Performance Indicators (KPIs) represent a minimum standard of care and provide a framework to improve and measure the quality and safety of care [22]. The Pediatric Rheumatology Care and Outcomes Improvement Network (PR-COIN) is an established North American learning health network (LHN) dedicated to improving outcomes and quality of care for children with juvenile idiopathic arthritis (JIA), the most common pediatric rheumatic disease [15,23,24,25]. PR-COIN was initially established in 2011 with 12 centers measuring and reporting performance on process and outcome quality measures for children and adolescents with JIA [24,26,27]. This LHN currently has 23 participating sites, with the registry containing data from over 13,500 patients [24]. PR-COIN has evolved since inception and now tracks 20 quality measures, adopting some from the initially established core set in 2011, and expanding quality measure categories to include outcome, process, balancing and data quality measures in 2019 [23,24,26]. This evolution and quality measure selection process is well-documented by Bingham et al. in 2023 [23]. Process of care indicators for JIA have also been established for quality improvement in Canada, with most KPIs consistent with the initial 2011 PR-COIN core set incorporating process measures of disease activity and safety monitoring [22,26]. With the extensive PR-COIN registry and relevance to the Canadian context, there is an established framework of KPIs with set performance goals available to evaluate and compare the quality and safety of a Canadian APP SCM in pediatric rheumatology. This will add valuable information to the currently lacking knowledge base regarding long-term sustainability and impact of an APP role [19,22,26].

In 2020, an APP SCM was implemented in a pediatric rheumatology center at McMaster Children’s Hospital in Hamilton, Ontario, Canada. The primary objective of this study was to identify the proportion of process KPIs attained in the long-term care of children with JIA followed in an APP SCM in pediatric rheumatology. The secondary outcome was to compare the APP SCM documented KPI frequency with the PR-COIN registry performance goals and KPI documentation at the Same Center and across the registry, representing usual care, to determine the quality and safety of care delivered in an APP SCM in pediatric rheumatology.

## 2. Methods

### 2.1. Ethics

The Hamilton Integrated Research Ethics Board (HiREB—#17728) provided ethical approval for this study. A waiver of consent was provided to complete the retrospective chart review.

### 2.2. Model of Care

The APP SCM was implemented in 2020 in a tertiary care children’s hospital in Hamilton, Ontario, Canada. Initially, one APP worked with two pediatric rheumatologists at 0.2 full time equivalent (FTE), expanding to 1.0 FTE with three pediatric rheumatologists in 2023. At the time of starting in the role, the APP was an experienced physiotherapist working for 20 years, 13 in rheumatology. Advanced training was undertaken in 2018–2019 through the “Advanced Clinician Practitioner in Arthritis Care” (ACPAC) program [11], an internationally recognized university-affiliated competency-based training program with standardized assessment for advanced practice/extended-scope clinicians [28].

**Figure 2:**
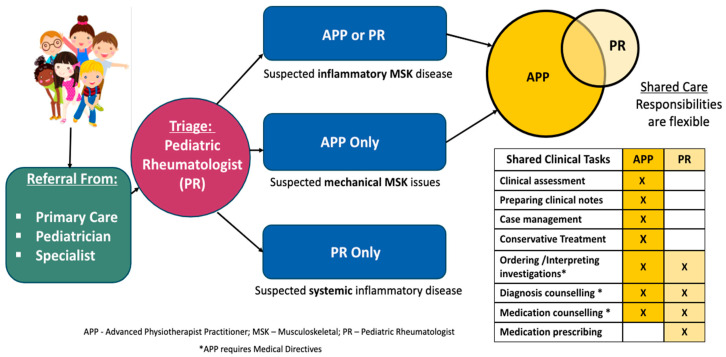
Advanced Physiotherapist Practitioner Shared-Care Model (APP SCM) in Pediatric Rheumatology.

In the APP SCM, new consults are triaged by a pediatric rheumatologist (PR) to the APP to be seen as the first point of care if the predominant reason for referral is an MSK complaint of any urgency (urgent to non-urgent) (Figure 2). Typical cases seen in the APP SCM include non-systemic JIA, chronic nonbacterial osteomyelitis, Lyme arthritis, and non-inflammatory MSK pain syndromes. Suspected complex systemic diseases (i.e., systemic lupus erythematosus, scleroderma, fever syndromes) are typically triaged for PR consult only, and, if seen in the APP SCM, these cases are transferred to the PR. At the initial consult, the APP completes a full comprehensive subjective and objective assessment, formulates an impression, and provides a diagnosis where possible. Medical directives are used for tasks beyond the scope of traditional physiotherapy and these may include ordering and interpreting laboratory investigations and diagnostic imaging (Magnetic Resonance Imaging, X-ray, Ultrasound, Computed Tomography), communicating a medical diagnosis (JIA and/or other rheumatic diseases), and initiating referrals to other specialties (i.e., orthopedics, dermatology, physiatry, chronic pain). The APP does not have medical directives to prescribe medication or to act as the “Most Responsible Practitioner”. Involvement from the PR varies according to the complexity of each case, with non-rheumatic disease cases requiring minimal involvement and newly diagnosed rheumatic disease cases requiring more involvement due to prescribing of medications or navigating unforeseen medical complexities. If a child is diagnosed with a rheumatic disease in the APP SCM, the APP becomes the primary point of contact for that child and family. All subsequent follow-up visits are booked with the APP for ongoing management.

### 2.3. Study Design

This retrospective longitudinal cohort study was conducted using electronic medical records (EMRs) between June 2022 and May 2024, inclusively. The onset of this 24-month period coincided with the implementation of the hospital’s new EMR system. The time period ended in May 2024, before initiating data extraction in June–July 2024, All APP SCM clinics during this time were identified. New consults concluding with a diagnosis of JIA, as defined by the International League of Associations for Rheumatology criteria, were reviewed [29]. Subsequent follow-up appointments in the APP SCM of these confirmed JIA cases were also reviewed.

### 2.4. Participants/Registry

During this time, the APP worked one clinic day per week for 15 months and three clinic days per week for 9 months yielding 168 clinic dates to review. The clinic averaged one to two new consults per clinic day. Approximately 20% of new consults seen in the APP clinic are diagnosed with a rheumatic disease and subsequently followed long-term [18]. Cases for EMR review of KPI documentation included all pediatric patients (<18 years old) with non-systemic JIA diagnosed and followed in the APP SCM during the study period. The APP does not manage patients with systemic JIA due to the medical complexity and lower MSK focus that is typical of this subtype. Cases were excluded if they did not have a rheumatic disease diagnosis, or if the diagnosis was a rheumatic disease other than non-systemic JIA.

Comparative data were obtained from the PR-COIN registry. This study was designed during the time of PR-COIN revisions. Documented KPI process measures for disease activity and safety monitoring were selected for data extraction to align with the initial process measures established by PR-COIN in 2011 (Table 1).

PR-COIN patients were included if they had active visit data between 1 June 2022 and 31 May 2024, regardless of day of diagnosis. Systemic JIA visits were excluded to be consistent with the APP typical caseload; however, all other JIA visits in this period were included. Patient registry data was separated into a Same Center (SC) group, representing usual care at the APP’s center with data from 3 rheumatologists, and all other active PR-COIN sites (15 centers) during that period.

### 2.5. Data Collection

Patient demographics and JIA disease characteristics were obtained from the EMR via retrospective chart review, including age at diagnosis of JIA, sex at birth, subtype of JIA and at each visit, time since diagnosis was recorded. KPIs were extracted according to PR-COIN’s initially established process KPIs for disease activity monitoring and safety monitoring [26] (Table 1). Disease activity monitoring KPIs included active joint count, pain, physician and Patient Global Assessment (PGA, PtGA), a composite clinical score (10-joint clinical Juvenile Disease Activity Score—cJADAS10), physical function (Childhood Health Assessment Questionnaire—CHAQ) and quality of life (QoL). The cJADAS is calculated by adding the PGA (0–10), PtGA (0–10), and number of joints with active arthritis (active joint count; 0 to a maximum of 10 joints) for a total possible score of 30 representing most severe disease activity [23]. Safety monitoring KPIs included screening for tuberculosis prior to starting a biologic DMARD, laboratory monitoring for DMARD toxicity, and uveitis screening per Heiligenhaus guidelines [30].

The access to care KPI was selected as “percentage of patients seen in yearly follow-up,” an established Canadian KPI and a newer PR-COIN quality measure [23]. Data were not available from the PR-COIN registry for this measure during the study period.

The frequency of KPIs documented during APP SCM clinic visits were first compared to PR-COIN’s recommended performance goals [26]. Data within the same two-year period, were also compared with the documented frequency of process KPIs from the PR-COIN registry for the SC and from 15 additional PR-COIN centers. Finally, outcomes for the disease activity KPIs were extracted for the APP SCM. Outcomes were not compared with the SC or the PR-COIN registry for several reasons. Between groups, disease duration was different; sample size was low in JIA subtypes where different outcomes are expected and availability of outcomes from the PR-COIN registry varied due to change in quality measure collection [23,24]. The KPI outcomes for children with JIA cared for in the APP SCM were still of interest as it provides important data to support the APP role’s long-term sustainability and relevance to future health care needs and therefore was included [19]. Outcome comparison was possible between groups by calculating cases reaching minimal disease activity during the study period, as PR-COIN collected this quality measure during the study period [23]. Although minimal disease activity has specific definitions depending on JIA subtype, cutoffs were defined as Pain ≤ 1 and cJADAS10 ≤ 3 for this study, as the sample size was too small to apply specific values to each subtype [31,32].

### 2.6. Data Analysis

Descriptive statistics were used to characterize the study population with continuous variables reported as means and standard deviations, and categorical variables summarized using frequencies and proportions. Due to the substantial imbalance in sample size between the PR-COIN group and the other two groups, statistical comparisons were limited to APP and SC groups when comparing minimal disease activity. Group differences were assessed using Kruskal–Wallis rank sum tests for continuous variables and Chi-square or Fisher’s exact tests for categorical variables, as appropriate. Statistical analyses of the PR-COIN data were conducted by the network statistician.

## 3. Results

The APP saw 33 unique patients with 1–8 visits each totaling 138 eligible visits. The SC saw 69 unique patients and PR-COIN saw 4031 for a total of 140 and 11,431 eligible visits, respectively.

### 3.1. Patient Demographics

Demographic and clinical characteristics of patients across comparison groups are summarized in Table 2. The mean age of APP patients was younger (9.3 years) compared with the SC and PR-COIN group visits (12.3 and 13.4 years, respectively). The sex distribution was similar across three groups. Mean time from diagnosis to each visit was markedly shorter in the APP group (mean 65 weeks) compared with SC (446 weeks) and PR-COIN (514 weeks). Given the APP SCM was new at study inception, all APP patients in this study were newly diagnosed during the study period. Regarding JIA subtypes, the APP group had higher proportions of polyarticular rheumatoid factor positive (RF+) subtype (9.1%) and psoriatic arthritis subtype (18%), while polyarticular RF- subtype was more prevalent in the SC (44%).

### 3.2. KPI Frequencies

Documentation of KPIs varied across the three groups, with all meeting and, in most cases, exceeding all PR-COIN performance goals for disease activity monitoring (Table 3). The SC had values minimally lower than the 80% performance goal for Pain, PtGA and cJADAS10 (78%, 76% and 74%, respectively). Assessment of functional ability using the CHAQ and QoL measures were the lowest across all groups, though APP (74% and 83%, respectively) and SC (73% and 82%) still met the performance goals of >70%.

All groups substantially exceeded the safety KPI performance goal for uveitis screening (40%). The APP met or exceeded the performance goals of the remaining safety KPIs. All nine cases that started biologics in the study period had the required TB screening. Of the 58 visits where DMARD lab screening was required, the APP documented this 93% of the time. Comparison was not possible for these safety KPIs as data were not available from the PR-COIN registry during the study period.

### 3.3. Clinical Outcomes

Table 4 summarizes clinical outcomes. Average time from day of diagnosis (usually first visit) to visit of data extraction was approximately 1 year (65 weeks). Most clinical outcomes indicated low disease activity. PtGA was higher than PGA with mean 2.24 (SD-2.29), scored from 0 to 10, with 0 indicating the best global assessment score. Mean composite score of cJADAS was 4.8 (SD-6.0), indicating minimal to moderate disease activity depending on JIA Subtype [31]. The proportion of patients attaining minimal disease activity was consistent between groups (Table 5). Minimal disease activity scores were statistically significant between APP and SC groups indicating APP outcomes met the current standards of care delivered by the SC.

## 4. Discussion

Dimensions of care identified by PR-COIN as important to improve quality of care for children with JIA, include efficacy, safety and timeliness [27]. These dimensions have translated into KPIs agreed upon by experts and are measurable in several domains, ultimately guiding improvement in care. The disease activity domain is intended to measure efficacy; the safety monitoring domain is intended to measure safety, and the access to care domain measures timeliness [26]. All groups generally met performance goals for KPIs, and the APP had documentation rates that were as good or better than the SC and PR-COIN [26]. The findings from this study suggest that targeted local initiatives, such as implementing an APP SCM, may enhance quality of care for children with JIA through comprehensive clinical documentation and adherence of established KPIs.

Disease activity KPIs were documented well above the performance goals (>80%) for all groups including the APP. The APP documented active joint count, PtGA, PGA and the composite disease activity score (cJADAS10) more than 95% of visits. A recent Canadian study at a tertiary care hospital evaluated similar disease activity KPIs in JIA care of 1360 visits with pediatric rheumatologists. Excellent documentation of joint assessment (99%) and pain (87%) were reported, with poor (≤15%) documentation of other clinical outcomes including PGA and cJADAS (cJADAS version not specified) [33]. A prospective study in the UK assessed disease activity measures of 1184 children with JIA followed by pediatric rheumatologists over a 14-year period. This study also reported excellent documentation of active joint count (92%) but otherwise did not meet PR-COIN performance goals (80%) for PGA (72%), PtGA (73%), Pain (73%) or cJADAS10 (57%) [34]. In our study, APP disease activity KPIs were compared to other PR-COIN centers, who, by association, are likely committed to quality improvement [25]. This may explain why all groups in this study had exceptional documentation practices leading to performance data that exceeded goals in almost all categories. PR-COIN-affiliated centers are potentially establishing the gold standard for the documentation of disease control measures in JIA care, with the APP SCM demonstrating adherence to these expected standards.

Functional ability (CHAQ) and QoL measures were documented less consistently across all groups, though the APP (74%;83%, respectively) and SC (73%;82%, respectively) groups met performance goals (>70%) and were comparable to the documented frequency of functional investigations in the UK (73%) [34]. The PR-COIN group had significantly lower documented frequencies for function (38%) and QoL (16%), similar to a Canadian study indicating functional status of children with JIA was documented in only 11% of cases [33]. While the APP SCM indicated a high level of documented patient-reported outcomes, this may reflect the implementation of systematic processes at the APP and SC’s center that facilitate the routine collection of patient-reported outcomes. Appropriate paper-based outcome measure packages are often prepared for each patient prior to clinic visits and these targeted efforts likely contribute to the assurance of this quality measure being completed during clinical visits.

The APP SCM demonstrated strong adherence to safety monitoring, including DMARD laboratory screening, TB screening, and uveitis screening. As new models of care become well-developed and reach optimal functionality, the overall impact and long-term sustainability must be evaluated [19]. At this stage, measurements must include adherence to quality and safety standards within the area of practice. While the field of advanced practice nursing has progressed to this evaluative stage, the literature on advanced practice physiotherapy remains largely concentrated on the initial phases of introduction and implementation, with only small single center studies pushing beyond these phases [21,35,36].

Improving access to care is a common justification for implementing innovative care models, which, in our study [7] was measured by ensuring children with JIA have at minimum one annual follow-up visit [22]. The APP SCM met this expectation in all of cases. The British Society for Paediatric and Adolescent Rheumatology (BSPAR) established standards of care in 2010, including an access to care standard that children be assessed by a pediatric rheumatology physiotherapist within 8 weeks of diagnosis [37]. When evaluated over a 2-year period among 10 pediatric rheumatology centers in the UK, only 45% of patients met this standard [37]. While this specific KPI is not currently adopted in Canada or by PR-COIN and was not directly measured in this study, all patients seen within the APP SCM had access to a physiotherapist. Although functioning in the role of an APP often precludes a full physiotherapy assessment and treatment, conservative physiotherapy-related advice is frequently provided during patient interactions [38]. This emphasizes an added value of physiotherapy expertise as a foundation for advanced practice roles in pediatric rheumatology [18].

Clinical outcomes in the APP group aligned with established JIA disease activity trajectories. With recent therapeutic advances, approximately 45% of children with JIA achieve minimal disease activity or remission within one year of diagnosis, increasing to 95% within five years [39,40]. Given that the average time from diagnosis in the APP group was approximately one year, the proportions of visits with pain ≤ 1 (62%) and cJADAS10 ≤ 3 (53%) indicate that cases followed in the APP group reflect expected JIA disease activity patterns [39].

Pediatric rheumatology remains underserviced across North America [40]. Models of care using APPs offer a strategy for maintaining quality, safety and efficacy of care for children with rheumatic diseases [15,17]. Physiotherapists with extended-scope training develop the expertise needed to fill gaps in the health care system by working within innovative care models [7]. In Canadian pediatric rheumatology, ACPAC-trained APPs have been shown to provide more rapid, prioritized, and consistent care, improving patient and family experiences [17]. This study demonstrates that care provided within an APP Shared-Care Model is safe, of high-quality, and is comparable to standard care, supporting the long-term stability of these roles.

### Limitations

Given this study was retrospective in nature, inherent limitations of retrospective studies must be considered. This study was completed at one center with one APP limiting the generalizability of results. Furthermore, the APP is also the primary investigator of this study, potentially contributing to unconscious or implicit bias when interpreting results. Steps were taken to have an independent, blinded research assistant complete data extraction and data analyses to decrease this potential bias. The immediate research team had several discussions to confirm interpretation of the data.

PR-COIN KPIs were selected for comparison as the APP’s hospital was a member of this North American Network at the time of the study. However, PR-COIN is composed of primarily pediatric rheumatology centers in the United States, with only two Canadian centers represented at the time of study. There is possible unknown bias when comparing data collected in two distinct health care systems, with Canada primarily operating under a public health care system and the US operating with a combined public/private system. Nevertheless, PR-COIN has the most robust database for comparison and as previously suggested, may currently be the gold standard for setting and attaining quality standards of care in JIA and was the optimal choice for this study.

JIA is a lifelong condition, and although a two-year study period is reasonable for assessing quality and safety through documentation, it remains relatively short for evaluating long-term outcomes. Safety was assessed through documentation of established KPIs; however, safety could also be assessed through rates of hospitalizations or serious adverse events. This was not addressed in this study. Future investigations should consider both a longer observation period and a broader definition of safety that includes additional clinically meaningful indicators.

The distribution of JIA subtypes differed between groups, with the APP group having a larger proportion of polyarticular RF positive and psoriatic subtypes, and no patients with oligo-extended or undifferentiated subtypes. When comparing rates of minimal disease activity, these differences may introduce bias due to variation in disease course across subtypes. In addition, only nine APP patients initiated biologic therapy during the study period, all of whom underwent TB testing. A larger cohort treated with biologics would improve the strength of this conclusion, specifically for this safety screening measurement.

Some of the data in PR-COIN may not fully reflect the performance of the individual centers as only certain data are considered critical elements for the registry to ease the process of data entry and assess the performance of particular JIA disease outcomes of interest by the network. This likely affected the PR-COIN CHAQ and other QoL measures presented in this study.

## 5. Conclusions

All providers in this study delivered safe, high-quality care for children with JIA. Care within the Canadian APP Shared-Care Model met international standards, with outcomes comparable to typical JIA disease trajectories [39].

This further supports the implementation of APPs in pediatric rheumatology to mitigate growing caseloads that are outpacing the workforce supply. This study also contributes to the growing international body of literature demonstrating the impact physiotherapists can safely have working in advanced practice/extended-scope roles. Next steps include replicating this study to investigate quality, safety and efficacy of care by other APPs in rheumatology settings.

## Figures and Tables

**Table 1:** PR-COIN process KPIs with performance goals for documentation in JIA Care [26].

Process KPIs	Definition	PR-COIN Performance Goal
**Disease Activity Monitoring**		
Active Joint Count	Percentage of patient visits where a joint count was conducted using a validated tool	≥80%
Assessment of arthritis-related pain	Percentage of patient visits where the patient was assessed for pain using any validated age-appropriate tool (when visits > 7 days apart)	≥80%
Practitioner Global Assessment (PGA) of disease activity	Percentage of patient visits where a PGA was completed using any validated tool	≥80%
(PtGA)	Percentage of patient visits where a PtGA was completed using any validated tool	≥80% *
Composite disease activity measurement	Percentage of patient visits with an assessment of disease activity using the cJADAS10	≥80% *
Assessment of functional ability (CHAQ)	Percentage of patient visits with a functional ability assessment using a validated tool (CHAQ)	≥70%
Quality of Life measurement	Percentage of patient visits with a measure of QoL using a validated tool (PedsQL)	≥70%
**Safety Monitoring**	**Percentage of patients**	
Uveitis Screening	with documented eye screening within the recommended timeline **	≥40%
DMARD Lab Screening	who received methotrexate and leflunomide and were monitored for toxicity by clinical laboratory methods	≥70%
TB screening	screened for TB prior to receiving a first course of therapy using a biologic DMARD	100%
**Access to Care**		
Yearly Follow Up	Percentage of patients who were seen at least once within a one-year period	PR-COIN Goal not established

CHAQ—Childhood Health Assessment Questionnaire; cJADAS10—clinical Juvenile Arthritis Disease Activity Score—10 joint; DMARD—Disease-Modifying Anti-Rheumatic Drug; KPIs—Key Performance Indicators; PedsQL—Pediatric Quality of Life; PGA—Practitioner Global Assessment; PR-COIN—Pediatric Rheumatology Care and Outcomes Improvement Network; PtGA—Patient Global Assessment; TB—tuberculosis. * Not explicitly stated by PR-COIN, however ≥80% was in line with expectations of other KPIs including PGA and joint count and was determined to be a reasonable goal for both the composite and Patient Global Score for this study. ** Uveitis screening timeline established by Heiligenhaus Guidelines [30].

**Table 2:** Patient demographic and disease characteristics across the groups (APP, SC, and PR-COIN).

Characteristics	APP (N = 33)	SC (N = 69)	PR-COIN (N = 4031)
Age, mean (SD)	9.3 (4.7)	12.3 (4.5)	13.4 (5.1)
Weeks from Diagnosis, mean (SD)	65 (65)	446 (168)	514 (198)
**Sex assigned at Birth, *n* (%)**			
Female	20 (61%)	45 (65%)	2813 (70%)
Male	13 (39%)	24 (35%)	1218 (30%)
**JIA Subtype**			
Oligoarticular, persistent	11 (33%)	16 (28%)	1274 (33%)
Oligoarticular, extended	0 (0%)	6 (11%)	272 (7.0%)
Polyarticular, RF−	8 (24%)	25 (44%)	943 (24%)
Polyarticular, RF+	3 (9.1%)	1 (1.8%)	206 (5.3%)
Psoriatic arthritis	6 (18%)	2 (3.5%)	382 (9.8%)
Enthesitis related arthritis	4 (12%)	6 (11%)	615 (16%)
Undifferentiated arthritis	0 (0%)	1 (1.8%)	198 (5.1%)
Unknown	1 (3%)	12 (17%)	141 (3.5%)

JIA—juvenile idiopathic arthritis; RF—rheumatoid factor; SD—standard deviation. Description of groups: APP—Advanced Physiotherapist Practitioner Shared-Care Model; SC—same center as APP representing Standard of Care (3 pediatric rheumatologists); PR-COIN—registry as a whole—15 centers (not including SC pediatric rheumatologists).

**Table 3:** Comparison of KPI documented frequencies of all visits in the study period between groups and against recommended PR-COIN performance goals.

	APP (N = 138)	SC (N = 140)	PR-COIN (N = 11,431)	PR-COIN Performance Goal (%)
**Disease Control KPIs**				
Active Joint Count	138 (100%)	149 (99%)	10,990 (90%)	≥80%
Arthritis-Related Pain	120 (87%)	118 (78%)	10,855 (89%)	≥80%
PGA	134 (97%)	148 (98%)	10,880 (89%)	≥80%
PtGA	131 (95%)	115 (76%)	11,173 (92%)	≥80% *
cJADAS10	131 (95%)	111 (74%)	10,109 (83%)	≥80% *
CHAQ	102 (74%)	110 (73%)	4564 (38%)	≥70%
QoL	115 (83%)	124 (82%)	1972 (16%)	≥70%
	**APP** **N Values Vary**	**SC** **(N = 140)**	**PR-COIN** **(N = 11,431)**	
**Safety KPIs**				
Uveitis Screening Heiligenhaus guidelines [30]	135/138 (98%)	118/140 (84%)	9593/11,431 (84%)	≥40%
DMARD Lab ** Screening	54/58 (93%)	Not Available	Not Available	≥70%
TB Screening ***	9/9 (100%)	Not Available	Not Available	100%
**Access to Care KPIs**				
Yearly Follow up ****	33/33 (100%)	Not Available	Not Available	No Goal

Description of Groups: APP—Advanced Physiotherapist Practitioner Shared-Care Model; SC—same center as APP representing Standard of Care (3 pediatric rheumatologists); PR-COIN—registry as a whole—15 centers (not including SC pediatric rheumatologists). * Not explicitly stated by PR-COIN, however ≥80% in line with expectations of other KPI’s including PGA and joint count and was determined to be a reasonable goal for both the composite and Patient Global Score. ** Percentage of patients who received methotrexate and leflunomide and were monitored for toxicity by clinical laboratory methods. *** Percentage of patients on biologic DMARDs and screened for TB prior to receiving a first course of therapy. **** Yearly follow up is an access to care KPI established for Canadian pediatric rheumatology standards [22]. CHAQ—Childhood Health Assessment Questionnaire; cJADAS10—clinical Juvenile Arthritis Disease Activity Score—10 joint; DMARD—Disease Anti-Rheumatic Drug; JIA—juvenile idiopathic arthritis; KPI—Key Performance Indicator; PGA—Practitioner Global Assessment; PtGA—Patient Global Assessment; QoL—Quality of Life; TB—tuberculosis.

**Table 4:** Disease activity KPI clinical outcomes for APP SCM.

	APP (N = 138)
Weeks from Diagnosis, mean (SD)	65 (65)
Active Joint Count, mean (SD)	1.46 (2.87)
Arthritis-Related Pain, mean (SD)	1.33 (2.17)
PGA, mean (SD)	1.08 (1.58)
PtGA, mean (SD)	2.24 (2.29)
cJADAS10, mean (SD)	4.8 (6.0)
CHAQ, mean (SD)	0.76 (0.18)
PedsQoL, mean (SD)	91 (18)

Description of Group: APP—Advanced Physiotherapist Practitioner Shared-Care Model. Clinical Outcome Assessment Tools: **Active Joint Count:** joint effusion or limitation of motion accompanied by heat, pain, or tenderness [29]; **Arthritis-Related Pain:** 10 cm VAS (0 = no pain); **PGA:** 10 cm VAS (0 = positive global assessment); **PtGA:** 10 cm VAS (0 = positive global assessment); **CJADAS10:** recorded as cJADAS10 = PGA + PtGA + Active Joint Count to a maximum of 10 (score range from 0 to 30); **CHAQ:** validated tool—0 represents no functional impact; 3 represents significant functional impact [32]; **QoL:** validated tool—PedsQL—Scoring is 0–100%, 100% represents excellent quality of life; CHAQ—Childhood Health Assessment Questionnaire; cJADAS—clinical Juvenile Arthritis Disease Activity Score; DMARD—Disease Anti-Rheumatic Drug; JIA—juvenile idiopathic arthritis; KPI—Key Performance Indicator; PGA—Practitioner Global Assessment; PtGA—Patient Global Assessment; PedsQoL—Pediatric Quality of Life.

**Table 5:** Visits With Minimal Disease Activity * Amongst Groups.

	APP (N = 138)	SC (N = 140)	PR-COIN (N = 11,431)	*p* Value **
**Arthritis-Related Pain ≤ 1**	85 (62%)	73 (52%)	5628 (49%)	<0.001
**cJADAS ≤ 3**	73 (53%)	77 (55%)	5915 (52%)	<0.001

Description of Groups: APP—Advanced Physiotherapist Practitioner Shared-Care Model; SC—same center as APP representing Standard of Care (3 pediatric rheumatologists); PR-COIN—Registry as a whole—15 centers (not including SC pediatric rheumatologists); cJADAS10—clinical Juvenile Arthritis Disease Activity Score—10 joint. * Minimal disease activity definition is dependent on JIA subtype [31,32]. For the purposes of this study, minimal disease activity was defined as cJADAS ≤3 and pain ≤1 and was applied to all subtypes. ** *p* Value only reflects statistical comparisons between the first two groups (APP and SC).

## Data Availability

The data presented in this study are available on request from the corresponding author due to privacy.

## Abbreviations

The following abbreviations are used in this manuscript:

ACPAC	Advanced Clinician Practitioner in Arthritis Care
APP	Advanced Physiotherapist Practitioner
BSPAR	British Society of Pediatric and Adolescent Rheumatology
CHAQ	Childhood Health Assessment Questionnaire
cJADAS10	Clinic Juvenile Arthritis Disease Activity Score 10 joint
DMARD	Disease-Modifying Anti-Rheumatic Drug
EMR	Electronic Medical Record
FTE	Full Time Equivalent
JIA	Juvenile Idiopathic Arthritis
KPI	Key Performance Indicators
LHN	Learning Health Network
MOC	Model of Care
MSK	Musculoskeletal
PEPPA	Participatory, Evidence-informed, Patient-centered Process for APN role development
PGA	Provider Global Assessment
PR	Pediatric Rheumatologist
PR-COIN	Pediatric Rheumatology Care and Outcomes Improvement Network
PtGA	Patient Global Assessment
QoL	Quality of Life
RF	Rheumatoid Factor
SC	Same Center
SCM	Shared-Care Model
